# No Evidence of Reduced Contrast Sensitivity in Migraine-with-Aura for Large, Narrowband, Centrally Presented Noise-Masked Stimuli

**DOI:** 10.3390/vision5020032

**Published:** 2021-06-21

**Authors:** Jordi M. Asher, Louise O’Hare, Paul B. Hibbard

**Affiliations:** 1 Department of Psychology, University of Essex, Colchester CO4 3SQ, UK; phibbard@essex.ac.uk; 2 Division of Psychology, Nottingham Trent University, Nottingham NG1 4FQ, UK; hare@ntu.ac.uk

**Keywords:** migraine with aura, psychophysics, contrast sensitivity, aura, cortical excitability, neural noise, spatial frequency

## Abstract

Individuals with migraine aura show differences in visual perception compared to control groups. Measures of contrast sensitivity have suggested that people with migraine aura are less able to exclude external visual noise, and that this relates to higher variability in neural processing. The current study compared contrast sensitivity in migraine with aura and control groups for narrow-band grating stimuli at 2 and 8 cycles/degree, masked by Gaussian white noise. We predicted that contrast sensitivity would be lower in the migraine with aura group at high noise levels. Contrast sensitivity was higher for the low spatial frequency stimuli, and decreased with the strength of the masking noise. We did not, however, find any evidence of reduced contrast sensitivity associated with migraine with aura. We propose alternative methods as a more targeted assessment of the role of neural noise and excitability as contributing factors to migraine aura.

## 1. Introduction

### 1.1. Background

The exact pathophysiology of migraine is still unclear, however the prevalence of photophobia and phonophobia (aversion to light and sound, respectively) that occur during the attack [1], or even in the absence of a headache [2], suggest that a migraine is a disorder of sensory processing [3]. Furthermore, visual discomfort to certain patterns and sensitivity to flickering light are commonly reported sensory triggers of migraine. Additionally, between 4% and 7% of people with migraine also experience sensory disturbances immediately preceding the onset of an attack [4]. These disturbances, or aura, while primarily visual, can occur in any sensory modality. Those with migraine aura typically experience hallucinations immediately before the onset of the headache [5], although aura can also occur without the headache [2]. Visual aura typically consists of expanding “fortification spectra” (shimmering zig-zag patterns) and a central scotoma (area of temporary blindness), although there are many other types of more complex aura hallucination [6].

Therefore, understanding the variances in sensory processing between those with migraine and those without may provide an insight to the underlying mechanisms of migraine. Compared to controls, people with migraine aura show heightened behavioural responses to sensory stimuli [7], increased EEG amplitude of the early visual components [8,9,10] and a higher susceptibility to phosphenes elicited by neurostimulation [11,12,13,14] between attacks. These findings are thought to represent an index of general cortical excitability [7,15,16], whereby there is a heightened response to incoming stimuli. Importantly, Brigo et al. [17]’s meta-analysis of susceptibility to phosphenes (as a proxy for cortical excitability) suggested the effect is specific for migraine aura, not migraine without aura. Behavioural responses have also been identified to be greater in migraine with aura than in those without aura [18,19]. There has been a suggestion that the two subtypes are distinct [20], although this is debated [21], and so the current study will focus on migraine with aura exclusively.

### 1.2. Contrast Sensitivity

**Figure 1:**
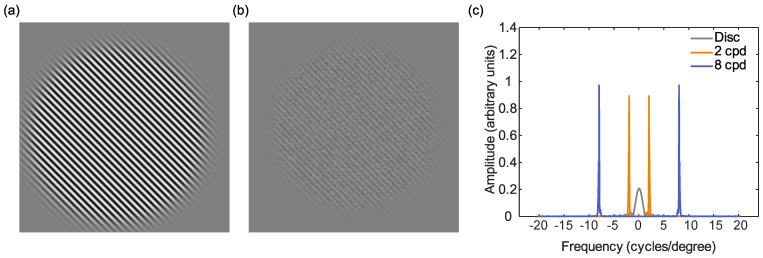
Schematic diagram of the stimuli used in the current experiment: (**a**) the 8 cpd target sine grating (exact spatial frequency content will vary as this is dependent on distance). (**b**) the target and Gaussian noise mask. (**c**) The Fourier transform of the stimuli, showing frequency plotted against amplitude (arbitrary units) for the disc used by Wagner et al. [18] (grey) the 2 cpd (orange) and the 8 cpd (blue) stimuli. The disc stimulus covers a wider range of spatial frequencies compared to the sine gratings that bias the visual system to preferred pathways.

Contrast sensitivity, the degree of contrast required to detect a stimulus, is often measured using sine-grating stimuli (see Figure 1) [22] as a function of their spatial and temporal frequencies. These stimuli are particularly useful for behaviourally investigating cortical hyperexcitability, which may occur as (i) a result of a reduced ability to ignore internal noise [22,23] or (ii) reduced inhibitory controls between neurons in the early visual processing areas [24]. Detection (or discrimination) of these gratings relies on the excitatory and inhibitory interactions between neurons. If there is a heightened response to incoming stimuli in those with migraine aura, it might be predicted that they should outperform controls on behavioural measures of contrast sensitivity. However, several studies have found reduced, rather than increased, contrast sensitivity when tested using static 4 cpd (cycles per degree) gratings [25,26,27,28].

Reduced sensitivity has also been found to flickering gratings, in particular 10–20 Hz flicker [29]. A reduction in contrast sensitivity was found only for low spatial frequency stimuli by Benedek et al. [30]. In contrast, Yenice et al. [31] found reduced contrast sensitivity for a range of spatial frequencies (1.5 to 18 cpd). This was a substantial effect, with a mixed (aura and without aura) migraine group showing just half the contrast sensitivity of a control group.

Other authors however find no difference in contrast sensitivity between migraine and control groups. Using a 3 cpd peak Gabor stimulus, McColl and Wilkinson [15] showed a trend towards poorer baseline contrast sensitivity in both migraine with and without aura groups, but this was not statistically significant. Although there was a reduction in performance in all groups from adding a 3 cpd grating mask (whether simultaneous with stimulus onset or asynchronous), there was no differential effect between the groups. Tibber et al. [32] also showed no differences between migraine aura, without aura and control groups for detecting a 4 cpd peak Gabor patch at cardinal or oblique angles. Asher et al. [33] found a small increase in contrast sensitivity in migraine with aura for centrally-presented 4 cpd Gabor patches, and Aldrich et al. [34] found no difference in contrast discrimination performance for 2.6 cpd Gabor stimuli presented against a 10% or 50% contrast pedestal.

Some studies have found no differences in overall contrast sensitivity [35], but losses in specific areas of the visual field when the stimuli flicker at 9 Hz or above [35]. This may result in smaller targets being missed. Additionally, this visual field loss seems to be worse just after the attack, then improves gradually, however this was in one observer reporting migraine without aura. Other studies have found differences in contrast sensitivity in the periphery only, at 12.5 degrees [36], and 10 degrees [37] eccentricity. Therefore, the current experiment will use large stimuli, covering a large area of the visual field, to allow for the potential deficits in specific areas of the visual field to be detected. Overall, research into contrast sensitivity in migraine with aura shows mixed results, and it is unclear what the reason for these differences might be [22].

### 1.3. Noise

Several researchers have found no differences in contrast sensitivity under optimal conditions, but that sensitivity is reduced when the stimuli are masked by adding external noise [18,38,39]. Wagner et al. [18] suggested that this was due to increased internal noise in response to a stimulus (neuronal response variability) in those with migraine aura.

### 1.4. Relation between Neural Noise, Contrast Sensitivity and Aura

The possibility that migraine with aura, in particular, is associated with an increase in neural noise, may help to understand the reason for the occurrence of the aura itself. The physiological correlate of migraine aura is thought to be a cortical wave of spreading depolarisation and depression [40]. This wave of neural excitation, followed by a period of reduced activity, has been used to account for the visual fortification (or zig-zag) patterns, and subsequent scotoma, experienced during a visual aura, respectively. Reaction-diffusion models of cortical spreading depolarisation and depression have been used to show how these self-sustaining patterns of activity can occur [41]. In these models, networks of neurons become susceptible to hallucinations through the balance of their excitatory and inhibitory interconnections (see [42] for a detailed review). An initial, localised occurrence of high activity is also required to trigger the spreading depolarisation. Increased levels of internal noise (additive or multiplicative) [18], or an increased gain on the responses to external stimuli [7,22] could both contribute to a greater susceptibility to the triggering of cortical spreading depolarisation and depression.

In summary, those with migraine aura have tended to show heightened sensitivity to visual stimuli [7], but poorer performance on contrast detection tasks, possibly due to an increased variability in neuronal responses to a stimulus (multiplicative internal noise) [18]. It could be the case that, while the increased levels of excitation result in greater overall activity, not all of this activity is specific to the stimulus. Such an increase in both signal and noise levels could reconcile the hyperexcitability found in migraine with aura with the fact that this does not lead to increased contrast sensitivity [7,22], but does predispose to visual aura.

### 1.5. Spatial Frequency—Which Spatial Scales of Processing Are Affected?

The spatial scale at which potential deficits in contrast sensitivity occur has not been the focus of much of the previous literature, with many studies using only one spatial frequency, e.g., [25,26,27,28].Where this has been looked at in detail [30], one study found that reduced contrast sensitivity in migraine with aura for static stimuli viewed at relatively high (photopic) luminance levels was confined to lower spatial frequencies, below 4 cycles/degree, and not found for spatial frequencies above this. Another study found reduced contrast sensitivity for all spatial frequencies between 1.5 and 18 cycles/degree [31] in migraine (not specifically migraine with aura). The work of Wagner et al. [18] and webster et al. [39], showing deficits in sensitivity only at high noise levels, used disc stimuli. These are low-pass (containing predominantly low spatial frequencies) but spatially localised (since small stimuli were used), and so are not well-suited to scale-space analysis. Therefore, in the current experiment, we used large, narrow band stimuli, with low and high spatial frequencies, in order to assess whether these noise-masking differences in contrast sensitivity occur for both coarse scale and fine scale mechanisms, respectively. In these stimuli, contrast energy is concentrated narrowly around a specific spatial frequency peak (Figure 1). Since contrast sensitivity across the visual field may be patchy, and associated especially with more peripheral vision [36,37], large stimuli were used. This also had the desirable effect of creating stimuli with a narrow spatial frequency bandwidth.

Analysis of the effects of spatial scale on contrast sensitivity can also, in some circumstances, be used to identify which visual pathways might be responsible for any deficits in processing. There are predominantly two visual areas responsible for the early encoding of visual information, processing information at different spatial scales. Around threshold levels of contrast, the magnocellular pathway is predominantly sensitive to coarse scale, low spatial frequencies below 1.5 cycles/degree. In contrast, the parvocellular system is predominantly sensitive to fine-grained information at spatial frequencies above this value [43,44,45]. Several authors have suggested that low-contrast stimuli favour the magnocellular pathway [46,47], based on single-cell recordings [48]. Other studies however have shown similar losses in contrast sensitivity in animal models for parvocellular lesions compared to magnocellular lesions [49], and that stimulus contrasts needed to elicit responses are similar for M and P cells in the owl monkey, but saturation levels are different [50].

By choosing appropriate stimuli, scale-space analysis can be used to some extent to investigate which of these two main pathways is the more affected. Although there are some reports of deficits in contrast sensitivity restricted to low spatial frequencies [30], consistent with a greater influence of the magnocelluar pathway, other studies have suggested that these effects occur at a range of frequencies [31], and are not associated exclusively with the magnocellular pathway [37]. The isolation of magnocellular from parvocellular processing using psychophysical techniques is difficult to achieve using only a single stimulus dimension, as in the current study, requiring the use of stimuli with a low spatial frequency, high temporal frequency, low contrast, low (scotopic) luminance, and adaptation to this luminance level [51]. The motivation for including spatial frequency in the current study was to assess how this affected contrast sensitivity and masking differences in migraine with aura [18,30,31,39], rather than specifically to assess the contributions of magnocellular and parvocelluar pathways to these effects.

### 1.6. The Current Study

Knowing the spatial scale at which any potential deficits occur is important for our understanding of the mechanisms involved in these differences. However, only one prior study has assessed this comprehensively, and this was for a mixed group of participants with and without aura [31]. Therefore, the aim of this study was to assess differences in contrast sensitivity at different masking noise levels, at low and high spatial frequencies exclusively in people with migraine with aura. To do this, contrast sensitivity was estimated at different noise levels for low and high spatial frequency sinusoidal grating stimuli. In line with previous research [18,52], we predict that deficits will only be found at high noise levels, not low noise levels. Since deficits in contrast sensitivity do not appear to be associated with a particular visual processing stream [37], we predict that these deficits could be for either fine, or coarse spatial scale, or both.

## 2. Methods

### 2.1. Participants

A total of 39 observers were tested. The categorisation of observers into groups was undertaken using the criteria of the Headache Classification Subcommittee of the International Headache Society [5]. All observers completed the experiment regardless of group. However, only data from individuals in the control or with aura group were included [17,18,19].

All observers were screened using a questionnaire by the experimenters (JA or PH). All observers had normal or corrected to normal vision. Inclusion as a control observer required no history of severe headaches, migraine, or aura. Migraine observers were tested interictally and were required to be free from migraine for 3 days either side of the day of testing. The data for 3 migraine observers were excluded as a result of experiencing an attack within 3 days of their testing day. After the classification process, there were 17 controls (9 females, mean age of 23.5 years) and 14 with migraine with aura (7 females, mean age of 31.7 years; see Table 1); 5 observers were excluded after being assessed as either migraine without aura, non-headache-free controls or migraine with aura not meeting inclusion criteria. No observers used prophylactic medication for migraine, and no observers were taking any substance that would affect cognition or perception. All experiments were conducted in accordance with the World Medical Association Declaration of Helsinki (2013) and were approved by the University of Essex ethics committee. All observers gave written, informed consent and received payment or course credit for their participation.

### 2.2. Apparatus

Stimuli were presented using a Sony Trinitron 2100 monitor with a screen resolution of 1280 × 1024 pixels and a vertical refresh rate of 100 Hz. The luminance response of the monitor was measured and calibrated using a Minolta LS-110 photometer. The luminance of the mid-grey background was 38.5 cdm${}^{2}$ and the maximum luminance of the monitor was 74 cdm${}^{-2}$. One pixel subtended 1.47 arc min. A Datapixx CRT Driver (Vpixx Technologies, Saint-Bruno, QC, Canada) was used to achieve 16-bit control of contrast levels. Stimuli were generated and presented using MATLAB and the Psychophysics Toolbox extensions [53,54,55]. Responses were made via the left and right arrow keys on a standard keyboard.

### 2.3. Stimuli

Stimuli were presented on a mid-grey background. The target stimuli were centrally presented sinusoidal gratings, with a spatial frequency of 2 or 8 cycles per degree, windowed with a circular aperture with a radius of 9 degrees, tapered with a Gaussian with a standard deviation of 0.98 degrees. The contrast of the target was manipulated: there were 10 contrast levels (0.05%, 0.01%, 0.02%, 0.3%, 0.45%, 0.5%, 0.75%, 1%, 2%, and 5% Michelson contrast). Each grating was presented at an orientation of $\pm 45^{\circ }$ from vertical, randomly selected with equal probability on each trial. In separate blocks of trials, static Gaussian white luminance noise with a standard deviation of 0. 3.5, 7.0, or 14.5 cdm${}^{-2}$ was used to mask the stimuli. This Gaussian noise was also tapered with the same window as the target stimulus.

### 2.4. Procedure

Observers were positioned at a viewing distance of 60 cm from the display, using a chin rest for support. The task consisted of a two-alternative-forced-choice (2AFC) procedure to report the orientation of the target grating. A central fixation cross was presented throughout the experiment. The stimulus, consisting of the target and mask, was presented for 360 ms. At the end of this time it was replaced by a blank grey screen and fixation cross while the participant responded. Participants completed either the 2 cycles/degree or 8 cycles/degree stimuli first, in random order. For each frequency, trials were blocked by noise level, and the order of presentation of these four blocks was also randomised. With each block, each of the 10 contrast levels was presented 20 times, given 200 trials per block. The order or presentation of these trials was randomised.

## 3. Results

**Figure 2:**
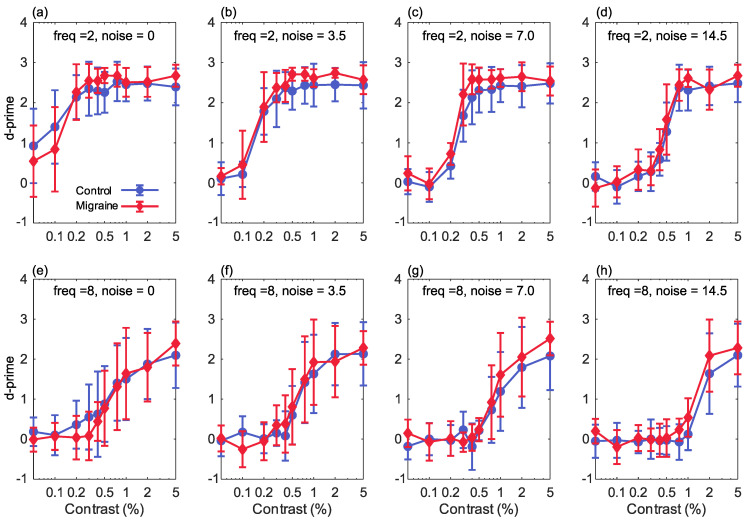
The results showing percentage Michelson contrast, spatial frequency against d’ (sensitivity) for the 2 cpd stimuli (top row) and the 8 cpd stimuli (bottom row), for increasing levels of Gaussian noise (n): (**a**,**e**) 0 noise (sigma of the Gaussian function = 0), (**b**,**f**) sigma is 3.5, (**c**,**g**) sigma is 7, (**d**,**h**) sigma is 14.5. Error bars show ±1 standard deviation.

The current study investigated the effect of increasing stimulus noise in contrast detection for a migraine with aura group in comparison with a control group. This was conducted both for low and high spatial frequency stimuli. For each contrast level in each condition, the percent correct was converted to $d^{{}^{\prime }}$, as a measure of each observer’s sensitivity to that stimulus (Figure 2). A 4-way, group × luminance contrast × noise level × spatial frequency, ANOVA was used to assess how sensitivity was affected by each of these factors. There was no main effect of group (*F*(1,29) = 1.605, *p* = 0.216, partial $\eta^{2}$ = 0.052), meaning that overall there was no difference in sensitivity between people with migraine with aura and the control group. There was a main effect of contrast (*F*(9,261) = 343.1, *p* < 0.001, partial $\eta^{2}$ = 0.922) reflecting the increase in correct responses with increasing stimulus contrast. There was a significant main effect of spatial frequency (*F*(1,29) = 524.0, *p* < 0.001, partial $\eta^{2}$ = 0.948), and a significant frequency-by-contrast interaction (*F*(9,261) = 62.40, *p* < 0.001, partial $\eta^{2}$ = 0.683), reflecting greater sensitivity to the lower spatial frequency. There was also a significant effect of noise (*F*(3,87) = 106.9, *p* < 0.001, partial $\eta^{2}$ = 0.787) and a significant noise-by-contrast interaction (*F*(27,783) = 16.728, *p* < 0.001, partial $\eta^{2}$ = 0.366), reflecting the reduction in correct responses with increasing noise level. A significant frequency-by-noise level interaction (*F*(3,87) = 10.28, *p* < 0.001, partial $\eta^{2}$ = 0.262) indicated a greater effect of noise at the higher spatial frequency.

There was a significant group-by-noise level interaction (*F*(3,87) = 2.751, *p* = 0.047, partial $\eta^{2}$ = 0.087). Sensitivity was greater in the migraine with aura group at the higher noise levels, but not at the lowest noise level. The group-by-contrast (*F*(2,261) = 1.564, *p* = 0.126, partial $\eta^{2}$ = 0.051) and group-by-frequency (*F*(1,29) = 0.891, *p* = 0.353, partial $\eta^{2}$ = 0.030) interactions were not significant.

There was a significant frequency-by-noise-by-contrast interaction (*F*(27,783) = 21.97, *p* < 0.001, partial $\eta^{2}$ = 0.420). The three-way interactions did not, however, indicate any differences between the two groups, since none of the group-by-frequency-by-contrast (*F*(9,261) = 1.55, *p* = 0.131, partial $\eta^{2}$ = 0.051), group-by-frequency-by-noise (*F*(3,87) = 0.778, *p* = 0.510, partial $\eta^{2}$ = 0.026) and group-by-noise-by-contrast (*F*(27,783) = 0.685, *p* = 0.765, partial $\eta^{2}$ = 0.023) interactions was not significant. The four-way group-by-frequency-by-noise-by-contrast interaction was also not significant (*F*(27,783) = 1.401, *p* = 0.087, partial $\eta^{2}$ = 0.046).

The contribution of migraine duration to noise-masked contrast detection in the migraine group was assessed using using a 3-way ANOVA (luminance contrast × noise level × spatial frequency) with migraine duration as a covariate. There was no significant main effect of duration (*F*(1,12) = 0.625, *p* = 0.444, partial $\eta^{2}$ = 0.050) and no significant 2-way, 3-way, or 4-way interactions between duration and frequency, noise or contrast.

To assess any differences in effects across scale, for each spatial frequency, $d^{{}^{\prime }}$ values were analysed using a 3-way contrast × noise × participant group mixed design ANOVA.

For the 2 cycles/degree stimuli, there was a main effect of contrast (*F*(9,261) = 423.9, *p* < 0.001, partial $\eta^{2}$ = 0.936), reflecting the increase in correct responses with increasing stimulus contrast. There was also a significant effect of noise (*F*(3,87) = 119.4, *p* < 0.001, partial $\eta^{2}$ = 0.805) and a significant noise-by-contrast interaction (*F*(27,783) = 28.73, *p* < 0.001, partial $\eta^{2}$ = 0.498), reflecting the reduction in correct responses with increasing noise level. A significant main effect of group (*F*(1,29) = 5.21, *p* = 0.030, partial $\eta^{2}$ = 0.152) and a significant group-by-contrast interaction (*F*(9,261) = 2.40, *p* = 0.012, partial $\eta^{2}$ = 0.077) were found, reflecting better overall performance in the migraine with aura group in comparison with the control group. The noise-by-group (*F*(3,87) = 1.63, *p* = 0.189, partial $\eta^{2}$ = 0.053) and noise-by-contrast-by-group (*F*(27,783) = 1.10, *p* = 0.331, partial $\eta^{2}$ = 0.037) interactions were not significant.

For the 8 cycles/degree stimuli, there was a main effect of contrast (*F*(9,261) = 136.4, *p* < 0.001, partial $\eta^{2}$ = 0.825), again reflecting the increase in correct responses with increasing stimulus contrast. There was a significant effect of noise (*F*(3,87) = 22.8, *p* < 0.001, partial $\eta^{2}$ = 0.440) and a significant noise-by-contrast interaction (*F*(27,783) = 9.88, *p* < 0.001, partial $\eta^{2}$ = 0.254), reflecting the reduction in correct responses with increasing noise level. For this spatial frequency, there was not a significant main effect of group (*F*(1,29) = 0.302, *p* = 0.587, partial $\eta^{2}$ = 0.010) or a significant group-by-contrast (*F*(9,261) = 1.249, *p* = 0.265, partial $\eta^{2}$ = 0.041) or group-by-noise (*F*(3,87) = 1.90, *p* = 0.135, $\eta^{2}$ = 0.062) interaction. The noise-by-contrast-by-group (*F*(27,783) = 1.05, *p* = 0.402, partial $\eta^{2}$ = 0.035) interaction was also not significant.

Performance was overall very similar between the two groups, and we did not find the expected reduction in sensitivity in the migraine with aura group at higher levels of noise. Performance was in fact slightly better in the migraine with aura group for low spatial frequency stimuli, although this difference was very small, as can be seen in Figure 2 (top row). The manipulations of the stimulus variables of contrast and noise level produced larger effect sizes (partial $\eta^{2}$ around 0.5 or above) than the group differences for the low spatial frequency stimuli (partial $\eta^{2}$ around 0.15 or below). On average, across all conditions, the increase in $d^{{}^{\prime }}$ value in the migraine with aura group for low spatial frequency stimuli, relative to the control group, was 0.155 (0.209).

## 4. Discussion

This study aimed to investigate contrast sensitivity under varying noise conditions in those with migraine with aura for two spatial frequencies, allowing for scale-space analysis. For the low spatial frequency stimuli, there was better performance in the migraine with aura group compared to the control group, for the higher levels of stimulus contrast. There were no differential effects of noise between groups. There were no group main effects or interactions for the high spatial frequencies.

### 4.1. Interpreting the Contrast Response Functions

The firing rate of each neuron depends on the contrast of the stimulus, where the firing rate increases above baseline as contrast increases and saturates as contrast intensifies. Plotting these responses typically shows a sigmoidal shape [56,57]. The contrast response function (CRF) illustrates the effect of contrast in visual processing. It has been suggested that detection of contrast can be improved by “raised attention” which increases the effective contrast. Based on the single cell recordings two models have been proposed to describe how attention and perception interact to improve contrast detection [58], *contrast gain* and *response gain*. Contrast gain is characterised by a shift in the psychometric function that is interpreted as a change to the threshold, where the threshold describes a response accuracy at chance level [59]. When directing attention to a specific location, sensitivity at that location is increased. Directed attention increases responses at low contrasts more than high contrasts [60]. This is consistent with an increase in physical or effective contrast, where performance saturates at higher contrast, and corresponds to the multiplication of contrast required to reach threshold. Response gain models predict that attention multiplies a neuron’s firing rate by a constant gain factor, whereby stimuli with increasing contrast will show an additive increase in firing rate [59,60] and are are characterised by a change in the slope and upper asymptote of the psychometric function [59].

**Figure 3:**
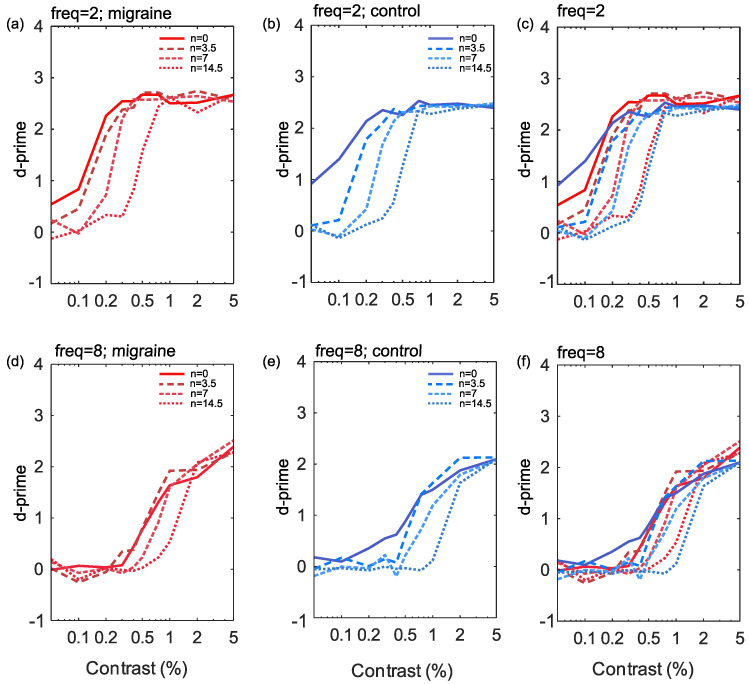
The results showing percentage Michelson contrast spatial frequency against d’ (sensitivity) for the 2 cpd stimuli (**a**–**c**) and the 8 cpd stimuli (**d**–**f**). Noise is plotted for each of the four levels independently for control (**b**,**e**) and migraine groups (**a**,**d**) and finally combined groups (**c**,**f**) by frequency.

The responses to increasing noise levels in the current study show a rightward shift in the CSF, (see Figure 3), indicative of reduced effective contrast, particularly for low spatial frequencies. There was no notable change to the slope or shift of the curve between migraine and control groups. While responses to high spatial frequency targets also display a tendency towards a rightward shift with increasing noise these were less pronounced than at low spatial frequencies.

Differences in sensitivity in migraine have been interpreted in previous studies using the perceptual template model [61]. This takes account of the efficiency of encoding, and the effects of additive and multiplicative noise on sensitivity. Changes in these parameters affect the slope of the psychometric function. Previous studies have focused not on the shape of the psychometric function, but on changes in threshold, and found that thresholds tended to increase only at high external noise levels [18,39]. In contrast, we found that the performance of control and migraine with aura groups was similar across all noise levels.

In general the slope of the curve was lower for high spatial frequency conditions (compared to low spatial frequency) with poorer performance at baseline (lower asymptote) as noise increased, possibly indicating reduced response gain [58]. Response gain has been linked to an overall increase in firing rate [59], suggesting the units are simply responding more overall for high spatial frequency targets with increasing external noise. However, again, these were similar across migraine and control groups.

To summarise, for low spatial frequency targets, there appears to be a multiplicative reduction in effective contrast at threshold as noise increases in both migraine and control groups. Contrary to previous work [18,19,39], there was no evidence of increased multiplicative internal noise in migraine compared to control groups in the current study. For high spatial frequencies, increasing noise slightly reduced effective contrast at threshold. However, there appears to primarily be reduced performance at baseline, increasing the contrast required at the lower asymptote. This could be indicative of increased response gain, which is linked to overall increase in overall firing rate [59]. However, once again, there is no evidence of a difference between those with migraine aura and controls.

### 4.2. Effects of Increasing Noise Levels

The low spatial frequency effect suggests that the differences in contrast sensitivity are more consistent with the contribution of the magnocellular system rather than the parvocellular system. However, these stimuli will not necessarily *isolate* the two pathways, but bias towards the favoured one [45]. The magnocellular system favours the lower spatial frequencies, and is thought to have a dominant role in processing transient visual stimuli. This might explain findings that those with migraine show increased performance for detecting briefly presented stimuli [62]. This is speculative, as in the current study all stimuli were presented at much longer intervals, and had a broader temporal frequency spectrum, than those required to see the benefit of briefly presented stimuli. To attempt to isolate the transient system, narrowband stimuli, and masks, with low spatial frequency, high temporal frequency, presented at scotopic luminance levels could be used [44,45,51,63,64]. This was beyond the scope of the current study, which focused on understanding how differences in contrast sensitivity in migraine with aura are influenced by spatial frequency and masking noise.

There was no differential group effect of increasing noise levels. It was expected that those with migraine with aura would show poorer contrast sensitivity at high noise levels, in line with previous research [18,52]. This was not found to be the case; our results do not show any evidence of increased additive or multiplicative internal noise in those with migraine aura on a contrast sensitivity task. This could be due to the choice of noise mask.

It is possible to measure internal noise using equivalent noise paradigms, which allow for estimations of internal noise, as well as the impact of adding external noise to the stimulus [65]. Performance at low noise levels is limited by the internal noise in the system itself, and the sampling efficiency. At high noise levels, the externally-added noise is much greater than the internal noise, rendering its effect negligible. The linear amplifier model (LAM) is one of the most straightforward ways of thinking about equivalent noise tasks. This model estimates the observer response as a linear combination of the contrast of the target, the noise internal to the system, and the noise associated with the target. The linear amplifier model assumes a linear response to increasing noise, which is not the case in contrast sensitivity tasks. In order to overcome this, a non-linear model can be fitted, with a gain control term. When using this non-linear model, it is not then possible to differentiate internal noise estimates from this gain control parameter. Therefore Baldwin et al. [66] suggested that pedestal noise masks could actually confound non-linear responses to the noise, from sources such as cross-channel suppression, rather than allowing for the estimation of internal noise. In the case of contrast sensitivity, a “zero-dimensional” noise mask can be added, instead of pedestal noise levels [67]. The “zero-dimensional noise” mask consists of contrast jitter of the target itself, rather than overlaying a separate white noise mask. By using the contrast jitter mask, rather than a pedestal mask, the possibility on non-linear effects of the mask can be differentiated, as it will limit effects such as cross-channel suppression.

The equivalent noise paradigm has been used in those with migraine, however this showed no differences in threshold performance between those with migraine and those without [68]. However, this was a mixed migraine group, rather than a purely migraine with aura group. The equivalent noise paradigm has also been applied by Tibber et al. [23] using a staircase method in the dimensions of motion, orientation, and size perception. They found a trend towards increased internal noise for motion perception in those with migraine, which was not statistically significant when corrected for multiple comparisons [23]. For motion, the high noise was added by changing the standard deviation of the dot trajectories, rather than adding additional “noise dots”. Again, the participants in this study were a mixed migraine group. It is possible that internal noise differences are specific to those experiencing migraine with aura, and so it would be good for future research to investigate this in an exclusively migraine with aura sample.

### 4.3. Migraine Duration

One reason for the lack of effects could be the duration of the migraine history of the participants. It is important to note that those with migraine with aura do not always show evidence of increased cortical excitability. Afra et al. [69] did not show a difference in baseline VEP (visually evoked potential) amplitude, although there was a facilitation of the response with repeating blocks of visual stimulation. Khalil et al. [8,70] found increased VEP amplitude, but only in those who had experienced migraine with aura for less than 10 years; those experiencing migraine with aura for longer than this showed a *reduced to normal* VEP amplitude. Khalil et al. [28] reported reduced contrast sensitivity, as well as P100 response amplitude (the positive peak in VEP at 100 ms) to 4 cpd gratings in those with migraine aura, and this related to the length of time the person had experienced migraine (accounting for age). The implication of these findings are that long-term repeated attacks may result in structural damage in the neural tissue that normalise the amplitude of the P100 response. Tibber et al. [32]’s participants had experienced migraine for around 15 years on average, which might explain the lack of findings. The participants in our study had experienced migraine with aura for between 2 and 44 years, with an average duration of around 14 years. 6 out of the 14 migraine participants had experienced migraine for more than 10 years, this may have diluted any effect. However, our analysis albeit with a small sample size, suggest there was no effect of migraine duration on contrast sensitivity.

### 4.4. Conclusions

In this study, we assessed whether contrast sensitivity deficits in migraine with aura would be evident only at high levels of external noise, and whether any such effects are influenced by the spatial frequency of the target stimuli. We conclude, however, that such estimates of contrast sensitivity using traditional stimuli and noise masks in those with migraine aura may not be the best tool to identify sensory processing differences between groups. Although contrast sensitivity provides an overall measure of visual sensitivity, there are many facets to the potential differences in people with or without aura that it is unable to capture. This is likely to account for the fact that previous findings are not robust [22], with some studies showing impaired contrast sensitivity [18,25,27,30,31,37,71,72], and others [15,32,33,34,39,73] showing no such deficits.

Contrast sensitivity is one of the most basic visual functions. It may be the case that differences in migraine aura are due to more complex mechanisms. For example, visual processing deficits across a range of conditions have been particularly associated with the dorsal processing stream [74], which depends on dynamic, low-frequency information. However, stimuli intended to isolate this "magnocellular function" are not precise in restricting processing to this channel [75]. Robust findings have tended to be for global motion stimuli (see [22] for a review), processed at higher stages of visual processing such as cortical area V5/MT [76]. This suggests that at this global stage of processing, rather than the earlier, local encoding stages assessed by contrast sensitivity measures, that will provide a clearer understanding of sensory differences in migraine. These studies have also suggested that differences might be particularly associated with a reduced ability to exclude noise [23], and that this might also be associated with an increased gain in response to external stimuli [7,22]. The use of zero-dimensional noise stimuli, rather than traditional contrast pedestals and noise masks, is better able to provide reliable measures of sensory noise and non-linear transduction of stimuli. Additive noise masks may invite other processes, such as cross-channel suppression [66], which may also differ in migraine.

The characteristics of individual participants, their long-term and short-term history of migraine and their migraine subtype are important considerations. Visual processing differences in migraine with aura are not necessarily shared by those without aura, for example [18]. Where differences are observed, they may be influenced by the length of time for which an individual has experienced migraine [70], and vary across the migraine cycle [77,78]. Together, these considerations suggest that measures of contrast sensitivity, at a single point in time, may not provide the most diagnostic assessment of sensory processing in migraine, and may account for the heterogeneous results that have been reported from such measures.

## Figures and Tables

**Table 1:** Migraine with aura observers’ reports of clinical features.

Observer	Sex	Age	Frequency (Per Month)	Duration (Years)	Prior Attack
OB4	M	22	1–3	7	8 days
OB7	F	20	1–3	12	>3 days
OB8	M	29	<1	6	3 weeks
OB10	M	20	1–3	5	1 week
OB14	M	20	<1	2	>3 days
OB18	M	24	1–3	6	>3 days
OB20	M	62	<1	3	2 months
OB21	F	40	1–3	15	1 month
OB22	F	50	3–10	37	10 days
OB26	F	19	<1	9	>3 days
OB35	M	22	1–3	8	>3 days
OB40	F	31	1–3	21	>3 days
OB41	F	59	3–10	44	4 days
OB42	F	26	1–3	16	1 month

## Data Availability

The data that support the findings of this study are openly available at https://osf.iokeajc/, accessed on 17 June 2021.

## Abbreviations

The following abbreviations are used in this manuscript:

cpd	cycles per degree
CRF	Contrast Response Function
EEG	Electroencephalogram
LAM	Linear Amplifier Model
VEP	Visual Evoked Potential
